# Supplementary material for: Effect of Sprouted Buckwheat on Glycemic Index and Quality of Reconstituted Rice
Source: Foods. 2024 Apr 10;13(8):1148. doi: 10.3390/foods13081148 (PMC11048801; doi:10.3390/foods13081148)
Supplement: Supplementary file 1 [file foods-13-01148-s001.zip › foods-2920436-supplementary.pdf]

Supplementary Material

Preparation process for recoSnstituted rice containing sprouted buckwheat

Lingtao Kang[a,b,c+], Jiaqian Luo[a,b,c+], Zhipeng Su[a,b,c], Liling Zhou[a,b,c], Qiutao Xie[b,c*],Gaoyang Li[a,b,c*]

[a]*Longping Branch, College of Biology, Hunan University, Changsha 410125, China*

[b]*Hunan Agricultural Product Processing Institute, Hunan Academy of Agricultural Sciences, Changsha 410125, China*

[c]*Hunan Provincial Key Laboratory for Fruits and Vegetables Storage Processing and Quality Safety, Changsha 410125, China*

Corresponding Authors

*E-mail addresses: lgy7102@163.com (G. Y. Li); 258222446@qq.com(Q.T.Xie)

[+]These authors contributed equally to this work

1. Experimental methods for formulation design and optimization

1.1 Mixing test design

The design of mixing experiments was carried out by setting constraints using Design-Expert 8.0.6 software. According to the results of the pre-test, due to the viscosity of purple sweet potato (D), sugar content is high. The content is too high will lead to the product being difficult to form, so its content percentage is fixed at 10%, wheat flour (A) and sprouted buckwheat flour (B) content percentage of the range of change are set to 20% ≤ A ≤ 50%. The range of variation of the black rice flour (C) content percentage is set to 10% ≤ C ≤ 30%. A+B+C = 90%, giving a total of 16 recipes. In this experiment, the percentage of raw materials was used as the independent variable, and the α-amylase inhibitory activity, resistant starch content, and sensory score of the product were used as the response values.

1.2 Extruded puffing process and parameters

Extrusion process： Cereal raw materials → Grinding (40 mesh sieve) → Moisture Adjustment → extrusion-puffing (reconstituted rice) → Microwave-puffing (instant reconstituted rice) → dry → Refrigerate and reserve.

Extrusion-puffing parameters： The raw materials were crushed and sieved and the moisture content was adjusted to 22%. The raw materials were extruded and expanded using a twin-screw extruder(LY70, Linyang Machinery, china) with a screw speed of 20.1 HZ, a temperature of 40.5°C in zone 1, 62.6°C in zone 2, and 68.7°C in zone 3.

Microwave-puffing parameters: expansion 1 min.

1.3 Determination of α-amylase inhibition rate and resistant starch content and sensory

evaluation

The inhibition rate of α-amylase was determined by referring to the Peng xi et al. method with minor modifications [1]. Take 5 g of the sample and add 0.1 mol/L PBS (pH 6. 9) and shake well, centrifuge at 3000 r/min for 10 min, and take the supernatant to be measured. Take 500 uL of the supernatant and add 500 uL of the α-amylase solution, incubate at 37°C for 10 min, then add 500 uL of 1% soluble starch, incubate at 37°C for 10 min, and then add 1 mL of DNS reagent, and terminate the reaction by boiling water bath for 5 min. Cooled to room temperature and diluted one-fold, the absorbance was measured at 540 nm using a Microplate reader (synergy H1, BIOTEK, USA). In the negative group, 500 uL of PBS was added to replace the supernatant. In the blank group, 1 mLPBS was added to replace the supernatant and α-amylase solution. The inhibition rate of α-amylase was calculated according to the following formula: inhibition rate (%) = $(A_1\text{-}A_2)$ - $(A_3\text{-}A_2)$ / $(A_1\text{-}A_2)$ × 100%. Where: A_1 is the negative group, A_2 is the blank group, and A_3 is the sample group.

Resistant starch content was determined according to the method of Goni et al. with minor modifications [2]. Weigh 200 mg of sample in a 50 mL centrifuge tube, digested by pepsin and cellulase successively, adjust the pH of the solution to 6.0, add thermostable α-amylase 1 mL (20 mg/mL), oscillate at 90 °C for 60 min, adjust the pH of the solution to 4.5 after cooling to room temperature, add amyloglucosidase 1 mL (20 mg/mL), oscillate at 60 °C for 60 min, and then centrifuge for 5 min at 8,000 r /min, discard the supernatant and repeat the water washing and centrifugation three times. To the precipitate, 2 mL of 2 mol/L KOH was added and shaken at room temperature for 30 min. pH was adjusted to 4.5, 1 mL of amyloglucosidase (20 mg/mL) was added, and the solution was shaken at 60°C for 60 min in a water bath. After cooling, centrifuge at 8000 r/min for 5 min, pour the

supernatant into a 100 mL volumetric flask, then repeat washing the precipitate with distilled water for 3 times, centrifuge, pour it into a volumetric flask, and then fix the volume to 100 mL with distilled water, shake it well, and then prepare for use. Glucose content was determined by the DNS method and then converted to the amount of resistant starch. Resistant starch yield was calculated according to the following formula: Resistant starch yield (%) = ($m_1 \times 0.9 \times 100$) /$m_2$. where m_1 is the mass of glucose (mg); m_2 is the mass of the sample (mg); 0.9 is the conversion coefficient between glucose and starch.

Sensory evaluation was carried out by a professionally trained 10-member tasting panel on the samples in four aspects, namely, morphology (25 points), color (20 points), taste (30 points), and flavor (25 points), respectively, and the specific scoring criteria are shown in **Table S1**.

Table S1 Sensory evaluation criteria

Items	Scoring criteria	Point
Morphology	Uniform texture, fine and smooth taste	16-25
	More homogeneous texture, finer and smoother taste	11-15
	Uneven texture and roughness	1-10
Color	Bright and even color, appetizing	16-20
	The color is brighter and more uniform and does not affect the appetite	11-15
	Poor color, seriously affecting appetite	1-10
Taste	Harmonized taste with no rawness, aftertaste or bitterness	16-30
	The palate is generally harmonious, with a slight rawness, aftertaste and bitterness	11-15
	Disjointed mouthfeel, with heavy rawness, aftertaste and bitterness	1-10
Flavor	Strong grain aroma with no undesirable flavors	16-25
	Has a light grain aroma with no undesirable flavors	11-15
	Odor or unpleasant taste	1-10

2. **Determination of optimal formulation for reconstituted rice**

2.1 Results of analysis with α-amylase inhibition as a response value

Statistical analysis, as well as multiple regression fitting of the experimental data for α-amylase inhibition in **Table S2**, was carried out using Design-Expert 8.0.6 software to obtain the regression equation:

Y_1=78.22A+53.22B−11.90C−180.89AB+6.80AC+46.31BC−690.38A^2BC+444.60AB^2C+1718.33A BC^2。The results of regression and ANOVA for α-amylase inhibition are shown in **Table S3**. The linear relationship between the regression model and the equation variables was highly significant, the lack of fit was not significant, and the model was well fitted to the test results.

From the regression equation Y_1, it can be seen that the response coefficients of the terms A, B, AC, and BC are all positive, indicating that wheat flour and sprouted buckwheat flour extracts as well as wheat flour-black rice flour and sprouted buckwheat flour-black rice flour mixed extracts can promote the effect of α-amylase inhibition, in which the response coefficient of wheat is larger, which reflects the strength of the three raw materials of α-amylase inhibition. Combined with **Table S3**, the effect of wheat flour-sprouted buckwheat flour co-action on α-amylase inhibition was highly significant ($p < 0.01$). The contour plot of the effect of raw material ratios on α-amylase inhibition rate is shown in **Fig.S1**. When the amount of wheat flour and black rice flour is more, and sprouted buckwheat flour is less, the α-amylase inhibition rate is about 50%; when the amount of wheat flour is more, and sprouted buckwheat flour and black rice flour is less, the α-amylase inhibition rate reaches 65%. The three raw materials in the compounding process on the α-amylase inhibition effect of synergistic effect, in which wheat flour plays a dominant role.

Table S2 Mixing test design and results

Test set	A<br>Sprouted buckwheat flour	B<br>wheat flour	C<br>black rice flour	D<br>purple sweet potato flour	R1<br>α-Amylase inhibition rate
1	20.000	50.000	20.000	10.000	43.826±2.08
2	30.000	30.000	30.000	10.000	54.016±1.54
3	40.000	20.000	30.000	10.000	48.877±0.66
4	27.500	42.500	20.000	10.000	44.946±0.25
5	50.000	30.000	10.000	10.000	42.428±0.54
6	30.000	50.000	10.000	10.000	24.094±1.55
7	20.000	40.000	30.000	10.000	31.224±0.42
8	35.000	35.000	20.000	10.000	43.644±1.69
9	42.500	32.500	15.000	10.000	32.098±1.06
10	20.000	50.000	20.000	10.000	47.290±0.38
11	40.000	20.000	30.000	10.000	47.820±1.14
12	25.000	50.000	15.000	10.000	43.512±1.85
13	50.000	30.000	10.000	10.000	33.589±2.85
14	20.000	40.000	30.000	10.000	33.314±2.19
15	30.000	50.000	10.000	10.000	25.892±1.65
16	40.000	40.000	10.000	10.000	21.335±1.42

Table S3 Analysis of variance (ANOVA) of α-amylase inhibition rate

Source	Sum of squares	df	Mean square	*F-ratio*	*Prob>F*
Model	1398.84	8	174.85	21.68	0.0003**
linear mixed model	490.17	2	245.09	30.39	0.0004**
AB	136.14	1	136.14	16.88	0.0045**
AC	4.53	1	4.53	0.56	0.4782
BC	2.7	1	2.7	0.34	0.5808
A^2BC	22.45	1	22.45	2.78	0.1392
AB^2C	12.93	1	12.93	1.60	0.2460
ABC^2	235.82	1	235.82	29.24	0.0010**
Residual	56.46	7	8.07		
Lack of fit	7.03	2	3.52	0.36	0.7172
Pure error	49.43	5	9.89		
	R^2 =0.9612			R^2_{Adj}= 0.9169	

Note: p > 0.05 is not significant; *: p < 0.05 is significant; **: p < 0.01 is highly significant, the same below.

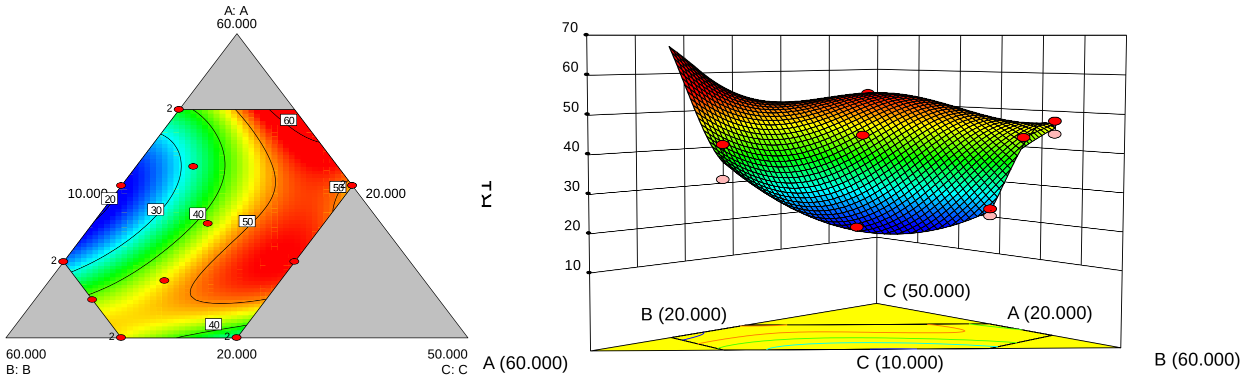

(a) Contour map (b) 3D Response surface map

Fig.S1. Influence of different proportions of extracts on *α*-amylase inhibition rate

2.2 Results of analysis with resistant starch content as response value

Statistical analysis as well as multiple regression fitting of the experimental data for resistant starch content in **Table S4** was carried out using Design-Expert 8.0.6 software and regression equations were obtained:

$Y_2=-1.28A+87.61B-638.67C-29.38AB+1360.30AC+1219.17BC-1163.68ABC+183.87AB(A-B)+720.62AC(A-C)-1103.64BC(B-C)$. The results of regression and ANOVA for resistant starch content are shown in **Table S5**. The linear relationship between the regression model and the equation variables was highly significant, and the lack of fit was not significant, indicating that the model was well fitted to the test results.

From the regression equation, Y_2 can be seen, B, AC, and BC response coefficients are positive, indicating that sprouted buckwheat flour content promotes resistant starch content, wheat flour-black rice flour, and buckwheat-black rice interaction can play a role in promoting the content of resistant starch, the size of the contribution of the wheat flour-black rice flour > sprouted buckwheat flour-black rice flour. It showed that sprouted buckwheat flour content plays an important role in resistant starch content, and the combined effect of wheat flour-black rice flour and sprouted buckwheat flour-black

rice flour significantly increased resistant starch content ($p < 0.01$). In addition, according to **Table S5**, three interaction terms were highly significant, indicating that the interaction of the three ingredients could significantly affect the resistant starch content. The contour plot of the effect of composite ingredient ratios on resistant starch content is shown in **Fig.S2**. When sprouted buckwheat flour accounted for a high percentage, wheat flour, and black rice flour accounted for a low percentage, the resistant starch content is around 41%. When the percentage of wheat flour and black rice flour is high and sprouted buckwheat flour is low, the resistant starch content reaches 42%. Three raw materials in the compounding process, sprouted buckwheat flour content, play a dominant role, and the three raw materials compounding have a synergistic effect on the content of resistant starch.

Table S4 Mixing test design and results

Test set	A Sprouted buckwheat flour	B wheat flour	C black rice flour	D purple sweet potato flour	R2 Resistant starch content
1	20.000	50.000	20.000	10.000	30.823±0.65
2	30.000	30.000	30.000	10.000	28.223±1.53
3	40.000	20.000	30.000	10.000	19.323±1.20
4	27.500	42.500	20.000	10.000	24.990±1.38
5	50.000	30.000	10.000	10.000	31.531±0.57
6	30.000	50.000	10.000	10.000	41.506±1.13
7	20.000	40.000	30.000	10.000	28.571±1.58
8	35.000	35.000	20.000	10.000	29.147±0.46
9	42.500	32.500	15.000	10.000	27.185±0.48
10	20.000	50.000	20.000	10.000	31.862±1.88
11	40.000	20.000	30.000	10.000	20.801±1.08
12	25.000	50.000	15.000	10.000	22.589±0.63
13	50.000	30.000	10.000	10.000	33.590±0.47
14	20.000	40.000	30.000	10.000	29.662±2.33
15	30.000	50.000	10.000	10.000	44.270±2.04
16	40.000	40.000	10.000	10.000	35.526±1.27

Table S5 Analysis of variance (ANOVA) of resistant starch content

Source	Sum of squares	df	Mean square	*F-ratio*	*Prob>F*
Model	664.97	9	73.89	32.7	0.0002**
linear mixed model	356.94	2	178.47	78.99	< 0.0001**
AB	2.75	1	2.75	1.22	0.3121
AC	113.03	1	113.03	50.03	0.0004**
BC	96.29	1	96.29	42.62	0.0006**
ABC	112.29	1	112.29	49.7	0.0004**
AB(A—B)	48.26	1	48.26	21.36	0.0036**
AC(A—C)	97.45	1	97.45	43.14	0.0006**
BC(B—C)	142.96	1	142.96	63.28	0.0002**
Residual	13.56	6	2.26		
Lack of fit	5.39	1	5.39	3.3	0.129
Pure error	8.17	5	1.63		
	R^2 =0.9800			R^2_{Adj}= 0.9501	

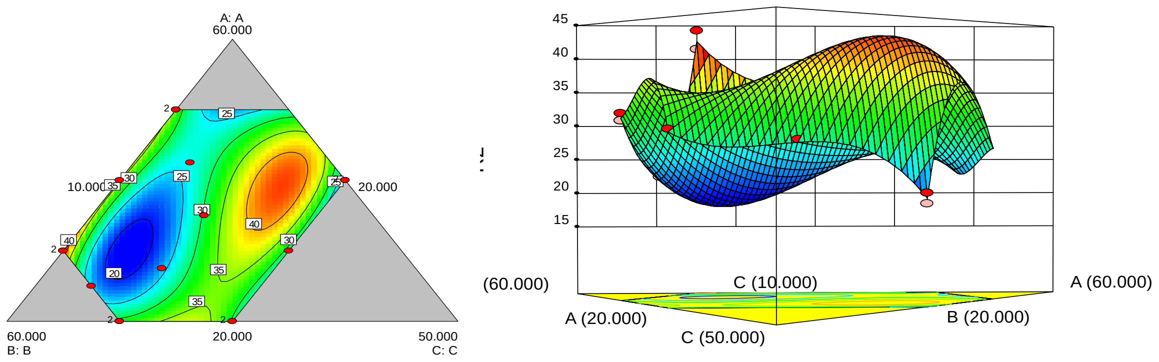

(a) Contour map (b) 3D Response surface map

Fig.S2. Effects of different ratios of raw material on the content of resistant starch

2.3 Results of analyses using sensory scores as response values

Statistical analysis as well as multiple regression fitting of the experimental data for the sensory scores in **Table S6** was performed using Design-Expert 8.0.6 software to obtain the regression equations:

Y_3=63.11A+77.73B−11.60C−8.27AB+166.51AC+114.84BC−165.44ABC+39.38AB（A−B）−138.60AC（A−C）−136.42BC（B−C）. The results of the regression and ANOVA for sensory scores are shown in **Table S7**, where the linear relationship between the regression model and the variables was highly significant and the lack of fit was not significant, indicating that the model was

well fitted to the test results.

From the regression equation Y_3, it can be seen that the response coefficients of A, B, AC, and BC are all positive, indicating that the use of wheat flour, sprouted buckwheat flour, and a mixture of wheat flour-black rice flour and sprouted buckwheat flour-black rice flour can play a role in promoting the sensory scores. The contribution of sprouted buckwheat flour > wheat flour and wheat flour-black rice flour > sprouted buckwheat flour-black rice flour, respectively. It showed that sprouted buckwheat flour played an important role, and the combined effect of wheat flour-black rice flour and sprouted buckwheat flour-black rice flour significantly increased the sensory scores ($p < 0.01$). The contour plot of the effect of composite ingredient ratios on sensory scores is shown in **Fig.S3**. When more black rice flour was used, and less wheat flour and sprouted buckwheat flour were used, the sensory score was higher, at about 79. The sensory score gradually decreased as the amount of black rice flour decreased.

Table S6 Mixing test design and results

Test set	A Sprouted buckwheat flour	B wheat flour	C black rice flour	D purple sweet potato flour	R3 Sensory score
1	20.000	50.000	20.000	10.000	70.000±2.83
2	30.000	30.000	30.000	10.000	78.900±2.16
3	40.000	20.000	30.000	10.000	79.600±1.32
4	27.500	42.500	20.000	10.000	69.500±3.12
5	50.000	30.000	10.000	10.000	69.500±3.16
6	30.000	50.000	10.000	10.000	70.200±2.58
7	20.000	40.000	30.000	10.000	72.800±1.99
8	35.000	35.000	20.000	10.000	72.600±2.54
9	42.500	32.500	15.000	10.000	68.300±2.50
10	20.000	50.000	20.000	10.000	69.700±2.06
11	40.000	20.000	30.000	10.000	78.400±1.61
12	25.000	50.000	15.000	10.000	68.500±2.07
13	50.000	30.000	10.000	10.000	68.400±2.28
14	20.000	40.000	30.000	10.000	74.100±2.42
15	30.000	50.000	10.000	10.000	67.200±2.08

16	40.000	40.000	10.000	10.000	68.500±3.18

Table S7 Analysis of variance (ANOVA) of sensory score

Source	Sum of squares	df	Mean square	*F-ratio*	*Prob>F*
Model	240.58	9	26.73	19.86	0.0008**
linear mixed model	209.56	2	104.78	77.85	< 0.0001**
AB	0.22	1	0.22	0.16	0.7014
AC	1.69	1	1.69	1.26	0.3049
BC	0.85	1	0.85	0.63	0.456
ABC	1.11	1	1.11	0.83	0.3987
AB(A—B)	2.22	1	2.22	1.65	0.2466
AC(A—C)	3.61	1	3.61	2.68	0.1528
BC(B—C)	2.18	1	2.18	1.62	0.2498
Residual	8.08	6	1.35		
Lack of fit	1.43	1	1.43	1.07	0.3477
Pure error	6.65	5	1.33		
	R^2 =0.9675			R^2_{Adj}= 0.9188	

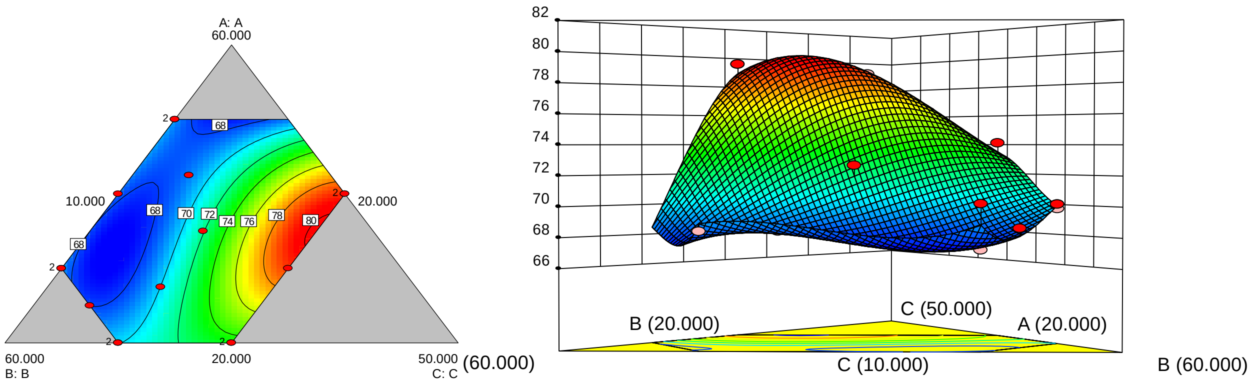

(a) Contour map (b) 3D Response surface map

Fig.S3. Effects of different ratios of raw material on sensory scores

2.4 Analysis results with weighted scores as response values

Statistical analysis as well as multiple regression fitting of the experimental data for the weighted scores in **Table S8** resulted in a regression equation:

$$Y_4=45.59A+67.70B-265.2C-80.75AB+611.88AC+551.99BC-545.25ABC+82.98AB(A-B)-488.68AC(A-C)-456.54BC(B-C)$$

. The results of the weighted score regression and ANOVA are shown in **Table S9**, the regression model was significant ($p < 0.05$), the lack of fit was not

significant, and the model was well fitted to the test results.

From the regression equation Y_4, it can be seen that the response coefficients of A, B, AC, and BC are all positive, indicating that the use of only wheat flour, sprouted buckwheat flour, and wheat flour-black rice flour, sprouted buckwheat flour-black rice flour interactively on the weighted scores have a promotional effect. The contribution of sprouted buckwheat flour > wheat flour and wheat flour-black rice flour > sprouted buckwheat flour-black rice flour, respectively. It showed that wheat flour and sprouted buckwheat flour played an important role, and the synergistic effect of wheat flour, black rice flour, and sprouted buckwheat flour-black rice flour significantly increased the weighted scores ($p < 0.01$). The contour plot of the effect of compound ingredient ratios on weighted scores is shown in **Fig.S4**. The three ingredient compounding acts synergistically on the weighted scores.

Table S8 Mixing test design and results

Test set	A<br>Sprouted buckwheat flour	B<br>wheat flour	C<br>black rice flour	D<br>purple sweet potato flour	R_4<br>weighted score
1	20.000	50.000	20.000	10.000	42.358
2	30.000	30.000	30.000	10.000	48.676
3	40.000	20.000	30.000	10.000	43.200
4	27.500	42.500	20.000	10.000	41.874
5	50.000	30.000	10.000	10.000	43.484
6	30.000	50.000	10.000	10.000	37.222
7	20.000	40.000	30.000	10.000	38.478
8	35.000	35.000	20.000	10.000	43.636
9	42.500	32.500	15.000	10.000	37.373
10	20.000	50.000	20.000	10.000	48.007
11	40.000	20.000	30.000	10.000	43.128
12	25.000	50.000	15.000	10.000	39.020
13	50.000	30.000	10.000	10.000	44.018
14	20.000	40.000	30.000	10.000	40.011
15	30.000	50.000	10.000	10.000	41.305
16	40.000	40.000	10.000	10.000	36.445

Table S9 Analysis of variance (ANOVA) of weighted score

Source	Sum of squares	df	Mean square	*F-ratio*	*Prob>F*
Model	166.78	9	18.53	4.34	0.044*
linear mixed model	24.61	2	12.31	2.88	0.1327
AB	20.78	1	20.78	4.87	0.0695
AC	22.87	1	22.87	5.36	0.0599
BC	19.74	1	19.74	4.62	0.0751
ABC	12.06	1	12.06	2.82	0.1438
AB(A—B)	9.85	1	9.85	2.31	0.1797
AC(A—C)	44.81	1	44.81	10.5	0.0177*
BC(B—C)	24.46	1	24.46	5.73	0.0538
Residual	25.62	6	4.27		
Lack of fit	0.010	1	0.010	0.002	0.9665
Pure error	25.61	5	5.12		
	R^2 =0.8668			R^2_{Adj}= 0.6671	

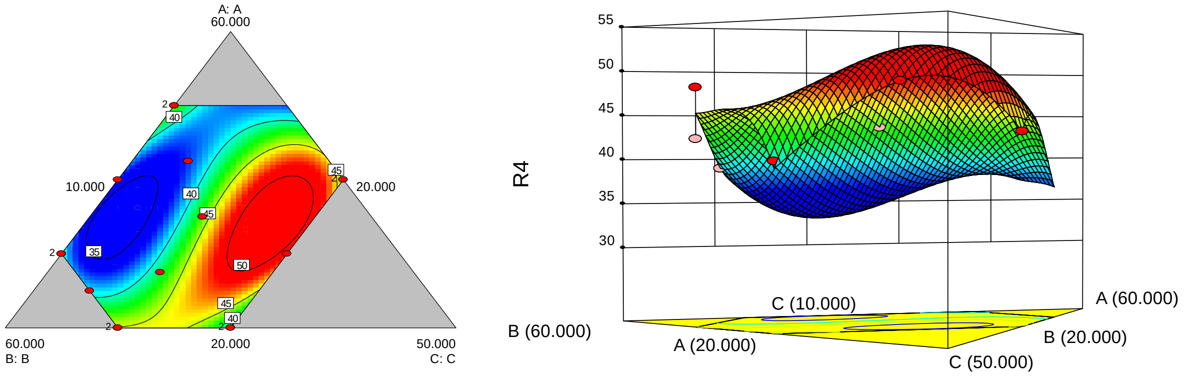

(a) Contour map (b) 3D Response surface map

Fig.S4. Effects of different ratios of raw material on weighted scores

Table S10 Flavor ingredient information of rice products

Class	Name	Rice Content (μg/g)	Instant rice Content (μg/g)	Reconstituted rice Content (μg/g)	Instant reconstituted rice Content (μg/g)
Alkanes	Decane	6.03±0.001	6.03±0.000	-	6.03±0.000
	Eicosane	39.17±0.000	-	39.179±0.004	39.2165±0.04
	Methylcyclooctane	-	3.64±0.000	-	-
	Dodecane	-	-	11.94±0.001	11.95±0.01
	n-Tetradecane	-	-	18.52±0.002	18.54±0.02
	n-Pentadecane	-	-	21.98±0.002	22.00±0.02
	n-Hexadecane	-	-	25.46±0.003	25.50±0.04
	Cyclopentadecan	-	-	-	21.43±0.000
	Nonadecane	-	-	-	35.71±0.000
	Undecane	-	-	-	0.48±0.014
	Phytane	-	-	39.19±0.006	-
	Cycloheptane	-	-	1.04±0.000	-
	n-Tridecane	-	-	15.18±0.002	15.19±0.016
Aldehydes	Nonanal	6.27±0.001	6.27±0.000	-	6.27±0.007
	Hexanal	1.19±0.002	1.19±0.000	-	1.19±0.001
	2,4-Nonadienal	-	14.94±0.000	5.52±0.000	14.94±0.000
	(E)-Hept-2-enal	-	2.04±0.001	2.05±0.001	-
	n-Octanal	-	3.92±0.000	3.92±0.001	3.92±0.000
	trans-2-Nonenal	-	5.87±0.000	-	-
	(E)-2-Octenal	-	-	20.55±0.000	20.57±0.009
	(Z)-2-Nonenal	-	-	5.87±0.001	-
	Decanal	-	-	8.81±0.001	-
	2-trans-Decenal	-	-	3.57±0.000	3.58±0.003
	Heptaldehyde	-	-	-	2.34±0.000
	Phenylacetaldehyde	-	-	-	2.05±0.003
	2,4-Decadienal	-	-	7.86±0.006	7.87±0.009
	trans-2,4-Decadienal	-	-	8.34±0.001	8.34±0.000
Naphthalenes	2-Methyl-1,2,3,4-Tetrahydronaphthalene	-	-	6.81±0.001	-
	1,5-Dimethyl-1,2,3,4-tetrahydro-naphthalin	-	-	9.67±0.000	9.69±0.02
	2,6-Dimethylnaphthalene	-	-	8.92±0.000	-
	1,5,7-Trimethyl-1,2,3,4-tetrahydronaphthalene	-	-	12.829±0.001	-
	1,2,3,4-Tetrahydro-4-isopropyl-1,6-dimethylnaphthalene	-	-	19.4±0.000	-
	2,3,5-Trimethylnaphthylene	-	-	11.96±0.000	11.98±0.019
	6-Ethyl-1,2,3,4-tetrahydronaphthalene	-	-	12.42±2.76	-
	2,5,8-Trimethyl-1,2,3,4-tetrahydronaphthalene	-	-	12.83±0.002	-
	5-Methyltetralin	-	-	6.81±0.001	-
	2-Methylnaphthalene	-	-	6.05±0.000	6.06±0.01
	1,4-dimethyl-1,2,3,4-tetrahydronaphthalene	-	-	9.68±0.000	9.67±0.001

Table S10 *Cont* 1.

Class	Name	Rice Content (μg/g)	Instant rice Content (μg/g)	Reconstituted rice Content (μg/g)	Instant reconstituted rice Content (μg/g)
Alcohols	N-methylphenylethanolamine	7.84±0.000	-	-	-
	α-Pinitol	-	-	8.43±0.005	-
	Spathulenol	-	-	23.86±0.003	-
	Isophytol	-	-	42.65±0.007	-
Esters	Dibutyl Phthalate	-	-	38.09±0.00	38.15±0.06
	Methyl linoleate	-	-	42.16±0.004	42.22±0.07
	l-ascorbyl dipalmitate	-	-	69.77±0.000	-
	Methyl hexadecanoate	-	-	-	36.23±0.000
	Methyl oleate	-	-	-	2.96±0.07
Olefin	Cinene	4.93±0.001	-	-	4.94±0.008
	D-Limonene	4.94±0.008	4.94±0.000	4.94±0.001	-
	Terpinene	-	-	5.00±0.001	-
Ketones	3-Octen-2-one	-	3.57±0.000	-	-
	6,10,14-Trimethyl-2-pentadecanone	-	-	35.70±0.000	35.76±0.06
	3-Nonen-2-one	-	-	5.87±0.001	5.88±0.006
Acids	Palmitic acid	-	-	32.74±0.03	32.77±0.003
	Stearic acid	-	-	39.69±0.000	39.74±0.04
	Oleic acid		-	-	42.68±0.000
Benzenes	1-isopropyl-2-methylbenzene	-	-	5.09±0.39	4.70±0.000
	o-Xylene	-	-	-	1.60±0.001
Indenes	1,2-Dimethyl-2,3-dihydro-1H-indene	-	-	6.8±0.001	-
	1,3,3-Trimethyl-1,2- dihydro-indene	-	-	9.66±0.001	-
Haloalkane	Dichloromethane	-	-	-	39.13±0.001
Pyrroles	1-(Phenylsulfonyl)Pyrrole	-	-	5.87±0.002	5.88±0.02
Sulfur-containing	Di-tert-dodecyl disulfide	-	-	62.61±0.007	-
Ethers	Vinyl isopropyl ether	-	-	-	0.57±0.006
Furans	2-Pentylfuran	-	5.51±0.000	5.51±0.000	5.52±0.006
Pyrazines	2-Methylpyrazine	-	-	-	0.81±0.001
Others	cedrol	24.37±0.004	-	-	-
	4,6,8-trimethylazulene	-	-	11.96±0.000	11.97±0.006

References:

[1] Peng, X.;Liu, K.;Hu, X.;Gong, D.;Zhang, G. Hesperetin-Cu(II) complex as potential α-amylase and α-glucosidase inhibitor: Inhibition mechanism and molecular docking. *Spectrochimica Acta Part A: Molecular and Biomolecular Spectroscopy* **2023**,290, 122301.http://10.1016/j.saa.2022.122301

[2] Goñi;, I.;García-Diz;, L.;Mañas;, E.;Saura-Calixto, F. Analysis of resistant starch: a method for foods and food products. *Food Chemistry* **1996**,56, 445-449
